# Supplementary material for: Germline-somatic JAK2 interactions are associated with clonal expansion in myelofibrosis
Source: Nat Commun. 2022 Sep 8;13:5284. doi: 10.1038/s41467-022-32986-7 (PMC9458655; doi:10.1038/s41467-022-32986-7)
Supplement: Supplementary file 1 — Supplementary Information [file 41467_2022_32986_MOESM1_ESM.pdf]

Supplementary Information

Germline-somatic *JAK2* interactions are associated with clonal expansion in myelofibrosis

Derek W. Brown, Weiyin Zhou, Youjin Wang, Kristine Jones, Wen Luo, Casey Dagnall, Kedest Teshome, Alyssa Klein, Tongwu Zhang, Shu-Hong Lin, Olivia W. Lee, Sairah Khan, Jacqueline B. Vo, Amy Hutchinson, Jia Liu, Jiahui Wang, Bin Zhu, Belynda Hicks, Andrew St. Martin, Stephen R. Spellman, Tao Wang, H. Joachim Deeg, Vikas Gupta, Stephanie J. Lee, Neal D. Freedman, Meredith Yeager, Stephen J. Chanock, Sharon A. Savage, Wael Saber, Shahinaz M. Gadalla, and Mitchell J. Machiela

Supplemental Table 1. Myelofibrosis population characteristics by analytic subset

Characteristic	Full CIBMTR Cohort	GWAS Cases	PacBio Sequencing	mCA Calls	Measured Telomere Length
N	937	827	924	933	916
Sex, N (%)					
Male	544 (58.06)	486 (58.77)	539 (58.33)	542 (58.09)	528 (57.64)
Female	393 (41.94)	341 (41.23)	385 (41.67)	391 (41.91)	388 (42.36)
Age at transplant, Mean (SD)	56.92 (10.40)	57.41 (9.68)	56.87 (10.43)	56.89 (10.40)	56.91 (10.46)
DNA source, N (%)					
Whole Blood	863 (92.10)	761 (92.02)	853 (92.32)	860 (92.18)	843 (92.03)
PBMC	74 (7.90)	66 (7.98)	71 (7.68)	73 (7.82)	73 (7.97)
Genetic Ancestry, Mean (SD)					
EUR	0.91 (0.22)	0.99 (0.03)	0.91 (0.22)	0.91 (0.23)	0.91 (0.23)
AFR	0.05 (0.16)	0.01 (0.02)	0.05 (0.16)	0.05 (0.16)	0.05 (0.17)
ASN	0.03 (0.16)	0.00 (0.01)	0.03 (0.16)	0.03 (0.16)	0.03 (0.16)
Genetic Ancestry Category, N (%)					
EUR	835 (89.11)	827 (100.00)	826 (89.39)	832 (89.17)	815 (88.97)
AFR	18 (1.92)	0 (0.00)	18 (1.95)	18 (1.93)	18 (1.97)
ASN	22 (2.35)	0 (0.00)	22 (2.38)	22 (2.36)	22 (2.40)
AFR and EUR	28 (2.99)	0 (0.00)	27 (2.92)	28 (3.00)	28 (3.06)
ASN and EUR	21 (2.24)	0 (0.00)	19 (2.06)	21 (2.25)	20 (2.18)
Other	13 (1.39)	0 (0.00)	12 (1.30)	12 (1.29)	13 (1.42)
Myelofibrosis Type, N (%)					
Primary	645 (68.84)	569 (68.80)	634 (68.61)	642 (68.81)	626 (68.34)
Secondary	292 (31.16)	258 (31.20)	290 (31.39)	291 (31.19)	290 (31.66)
Post-polycythemia vera[a]	136 (46.58)	119 (46.12)	135 (46.55)	136 (46.74)	135 (46.55)
Post-essential thrombocythemia[a]	156 (53.42)	139 (53.88)	155 (53.45)	155 (53.26)	155 (53.45)
DIPSS at Transplant, N (%)					
Low	73 (7.79)	64 (7.74)	72 (7.79)	73 (7.82)	73 (7.97)
Intermediate 1	216 (23.05)	189 (22.85)	212 (22.94)	215 (23.04)	212 (23.14)
Intermediate 2	246 (26.25)	221 (26.72)	245 (26.52)	245 (26.26)	239 (26.09)
High	9 (0.96)	8 (0.97)	9 (0.97)	9 (0.96)	8 (0.87)
Missing	393 (41.94)	345 (41.72)	386 (41.77)	391 (41.91)	384 (41.92)
Time from diagnosis to transplant (months), Mean (SD)	63.51 (82.14)	64.27 (83.26)	63.47 (82.37)	63.49 (82.26)	64.24 (82.80)

[a]Percentages are out of secondary myelofibrosis cases

Supplemental Table 2. Magnitude and strength of association for myelofibrosis susceptibility loci stratified by primary and secondary myelofibrosis status

								**Overall**		**Primary Myelofibrosis**		**Secondary Myelofibrosis**	
**Region**	**Top SNP**	**Nearby gene**	**Position (hg38)**	R^2	**Ref**	**Risk**	**RAF**	**OR (95% CI)**	**p-value**	**OR (95% CI)**	**p-value**	**OR (95% CI)**	**p-value**
3q25.33	rs201009932	*IFT80*	160368930	0.962	T	TA	0.01	5.78 (3.67, 9.11)	4.06×10^{-14}	4.70 (2.73, 8.11)	2.67×10^{-8}	9.05 (4.02, 20.38)	1.05×10^{-7}
4q24	rs1548483	*TET2*	104828738	0.951	C	T	0.04	2.27 (1.71, 3.01)	1.42×10^{-8}	2.30 (1.62, 3.25)	2.81×10^{-6}	2.17 (1.34, 3.51)	1.66×10^{-3}
5p15.33	rs7705526	*TERT*	1285859	0.998	C	A	0.34	1.65 (1.48, 1.84)	7.62×10^{-19}	1.54 (1.35, 1.76)	2.11×10^{-10}	1.94 (1.59, 2.36)	4.54×10^{-11}
6p21.32	rs28442287	*HLA-DRB9*	32658230	0.999	T	C	0.89	1.63 (1.38, 1.93)	9.34×10^{-9}	1.59 (1.30, 1.95)	5.27×10^{-6}	1.71 (1.27, 2.31)	4.66×10^{-4}
9p24.1	rs7851556	*JAK2*	5022807	0.970	C	T	0.31	2.39 (2.13, 2.68)	5.75×10^{-51}	2.08 (1.81, 2.39)	4.95×10^{-25}	3.19 (2.62, 3.89)	1.39×10^{-30}
17p13.1	rs78378222	*TP53*	7668434	1.000	T	G	0.01	4.47 (2.75, 7.27)	1.56×10^{-9}	3.48 (1.83, 6.63)	1.50×10^{-4}	6.70 (3.19, 14.06)	4.89×10^{-7}

R^2= imputation quality score in overall GWAS

Ref= reference allele

Risk= risk allele

RAF= risk allele frequency in overall GWAS

OR (95% CI)= myelofibrosis association odds ratio and 95% confidence interval adjusted for significant ancestry principal components

p-value= two-sided logistic regression

Supplemental Table 3. Magnitude and strength of association for myelofibrosis susceptibility loci stratified by secondary myelofibrosis type

						Post-Polycythemia Vera Myelofibrosis[a]		Post-Essential Thrombocythemia Myelofibrosis[b]	
**Region**	**Top SNP**	**Nearby gene**	**Position (hg38)**	**Ref**	**Risk**	**OR (95% CI)**	**p-value**	**OR (95% CI)**	**p-value**
3q25.33	rs201009932	*IFT80*	160368930	T	TA	5.12 (1.70-15.42)	3.70×10^{-3}	12.72 (4.01-40.28)	1.54×10^{-5}
4q24	rs1548483	*TET2*	104828738	C	T	4.00 (1.83-8.73)	5.04×10^{-4}	1.52 (0.82-2.82)	1.82×10^{-1}
5p15.33	rs7705526	*TERT*	1285859	C	A	2.13 (1.59-2.84)	2.87×10^{-7}	1.73 (1.32-2.26)	7.52×10^{-5}
6p21.32	rs28442287	*HLA-DQB1-AS1*	32658230	T	C	2.07 (1.32-3.26)	1.67×10^{-3}	1.45 (0.97-2.18)	7.21×10^{-2}
9p24.1	rs7851556	*JAK2*	5022807	C	T	5.31 (4.02-7.00)	4.06×10^{-32}	1.82 (1.36-2.42)	4.51×10^{-5}
17p13.1	rs78378222	*TP53*	7668434	T	G	19.89 (6.21-63.72)	4.82×10^{-7}	3.20 (1.22-8.38)	1.78×10^{-2}

[a]Analysis performed in 119 cases and 595 controls

[b]Analysis performed in 139 cases and 695 controls

Ref= reference allele

Risk= risk allele

OR (95% CI)= myelofibrosis association odds ratio and 95% confidence interval adjusted for significant ancestry principal components

p-value= two-sided logistic regression

Supplemental Table 4. *JAK2*^{V617F} mutation status by *JAK2* genotype (rs7851556) status

	rs7851556 Genotype			
	CC	TC	TT	p-value[a]
N	295	368	261	
JAK2^{V617F} mutation status				9.41×10^{-14}
Mutation	150 (50.85)	202 (54.89)	210 (80.46)	
No Mutation	145 (49.15)	166 (45.11)	51 (19.54)	

[a]Chi-square test

Supplemental Table 5. Germline haplotypes by *JAK2*^{V617F} mutation status

	JAK2^{V617F} mutation status			
	Mutation	No Mutation	Total	p-value[a]
N	562	1,286	1,848	
Germline Haplotype				
GGC	370 (65.84)	384 (28.86)	754	1.23×10^{-26}
TCT	187 (33.27)	887 (68.97)	1074	2.25×10^{-22}
GCC	4 (0.71)	6 (0.47)	10	0.5044
TGC	1 (0.18)	6 (0.47)	7	0.6831
CCT	0 (0.00)	1 (0.08)	1	1
GCT	0 (0.00)	1 (0.08)	1	1
TCC	0 (0.00)	1 (0.08)	1	1

[a]Two-sided binomial test

GGC= Germline risk haplotype

N= Number of germline haplotypes

Supplemental Table 6. Identified individuals with *JAK2*^{V617F} mutations potentially acquired on both germline haplotypes

		Original Sequencing		Resequencing	
Subject ID[a]	Germline Haplotype	Haplotype Reads	*JAK2*^{V617F} Mutation Count[b]	Haplotype Reads	*JAK2*^{V617F} Mutation Count[b]
1	GGC	4,067	128 (3.15)	10,683	353 (3.30)
1	TCT	5,223	2,318 (44.38)	14,424	5,991 (41.53)
2	GGC	183	77 (42.08)	1,572	1,005 (63.93)
2	TCT	5,484	2,803 (51.11)	25,398	12,342 (48.59)
3	GGC	379	135 (35.62)	1,653	683 (41.32)
3	TCT	10,698	157 (1.47)	28,923	693 (2.40)
4	GGC	3,286	226 (6.88)	9,479	804 (8.48)
4	TCT	5,086	4,194 (82.46)	14,691	12,197 (83.02)
5	GGC	3,368	167 (4.96)	10,619	254 (2.39)
5	TCT	3,076	199 (6.47)	11,274	843 (7.48)

[a]Subject ID 2 was diagnosed with post-polycythemia vera myelofibrosis, all other subjects were diagnosed with primary myelofibrosis

[b]Percentage of germline haplotype reads carrying the mutation

Supplemental Table 7. mCA enrichment over myelofibrosis susceptibility loci[a]

Region	Top SNP	Nearby gene	Position (hg38)	MF mCAs, N(%)	UKBB mCAs, N(%)	p-value[b]
3q25.33	rs201009932	*IFT80*	160368930	26 (3.06)	24 (0.056)	5.55×10^{-36}
4q24	rs1548483	*TET2*	104828738	24 (2.82)	25 (0.059)	4.34×10^{-32}
5p15.33	rs7705526	*TERT*	1285859	6 (0.71)	7 (0.016)	9.12×10^{-9}
6p21.32	rs28442287	*HLA-DRB9*	32658230	13 (1.53)	18 (0.042)	1.80×10^{-16}
9p24.1	rs7851556	*JAK2*	5022807	364 (42.82)	44 (0.104)	5×10^{-324} [c]
17p13.1	rs78378222	*TP53*	7668434	30 (3.53)	38 (0.089)	2.94×10^{-37}

[a]Myelofibrosis cases (N= 850) age (within 5-years) and sex-matched to UK Biobank cancer-free controls (N= 42,500)
[b]Two-sided binomial test
[c]Lowest estimated p-value available in R
mCA= mosaic chromosomal alteration
MF= Myelofibrosis
UKBB= UK Biobank

Supplemental Table 8. mCA event type over 9p24.1 by *JAK2* genotype (rs7851556) status

	rs7851556 Genotype			
	CC	TC	TT	p-value[a]
N	297	373	263	
mCA Status Over 9p24.1				2.28×10^{-19}
Normal	207 (69.70)	254 (68.10)	94 (35.74)	
Any	90 (30.30)	119 (31.90)	169 (64.26)	
Gain	10 (11.11)	30 (25.21)	8 (4.73)	
Loss	6 (6.67)	24 (20.17)	6 (3.55)	
CNLOH	74 (82.22)	65 (54.62)	155 (91.72)	

[a]Chi-square test between Normal and Any mCA status
mCA= mosaic chromosomal alteration
CNLOH= copy neutral loss of heterozygosity

Supplemental Table 9. *JAK2*^{V617F} mutation status by mCA status spanning 9p24.1

	mCA over 9p24.1			
	Yes	No	Total	p-value[a]
N	374	550	924	
JAK2^{V617F} mutation status				4.37×10^{-80}
Mutation	366 (97.86)	196 (35.64)	562 (60.82)	
No Mutation	8 (2.14)	354 (64.36)	362 (39.18)	

[a]Chi-square test

mCA= mosaic chromosomal alteration

Supplemental Table 10. mCA enrichment over myeloproliferative neoplasm driver mutation position[a]

Region	Gene	Position (hg38)	MF mCAs, N(%)	UKBB mCAs, N(%)	p-value[b]
1p34.2	*MPL*	43337818-43354466	31 (3.65)	60 (0.141)	6.50×10^{-33}
19p13.13	*CALR*	12938609-12944489	25 (2.94)	28 (0.066)	1.36×10^{-32}

[a]Myelofibrosis cases (N= 850) age (within 5-years) and sex-matched to UK Biobank cancer-free controls (N= 42,500)

[b]Two-sided binomial test

mCA= mosaic chromosomal alteration

MF= Myelofibrosis

UKBB= UK Biobank

Supplemental Table 11. Significant telomere-length associated germline genetic variants identified from Li et. al (2020) with myelofibrosis risk

						Telomere Length Association[a]		Association with Myelofibrosis[b]			
**Nearby gene**	**CHR**	**Position (hg38)**	**rsID**	**Ref**	**Alt**	**β**	**SE**	**β**	**SE**	**p-value**	**FDR[c]**
PARP1	1	226374920	rs3219104	A	C	0.0417	0.0064	0.0879	0.0731	0.2292	0.9926
TERC	3	169796797	rs10936600	T	A	0.0858	0.0057	0.0989	0.0633	0.1181	0.4082
MOB1B	4	70908630	rs13137667	T	C	0.0765	0.0137	0.0128	0.1609	0.9364	0.5030
NAF1	4	163127047	rs4691895	G	C	0.0577	0.0061	-0.1491	0.0657	0.0233	0.0005
TERT	5	1285859	rs7705526	C	A	0.0820	0.0058	0.4995	0.0563	7.62×10^{-19}	0.0000
TERT	5	1287079	rs2853677	A	G	0.0638	0.0055	0.2479	0.0547	5.84×10^{-6}	0.4461
CARMIL1	6	25480100	rs34991172	G	T	0.0608	0.0105	-0.1416	0.1127	0.2090	0.0342
PRRC2A	6	31619784	rs2736176	G	C	0.0345	0.0055	0.0164	0.0575	0.7755	0.4775
POT1	7	124914213	rs59294613	A	C	0.0407	0.0055	0.0205	0.0601	0.7328	0.4775
STN1 (OBFC1)	10	103916188	rs9419958	C	T	0.0636	0.0071	0.1013	0.0759	0.1821	0.7149
ATM	11	108234866	rs228595	A	G	0.0285	0.0050	-0.2258	0.0549	3.95×10^{-5}	0.0000
DCAF4	14	72938044	rs2302588	G	C	0.0476	0.0084	0.0663	0.0877	0.4492	0.8005
TERF2	16	69373083	rs3785074	A	G	0.0351	0.0056	-0.0488	0.0615	0.4271	0.2253
RFWD3	16	74646176	rs62053580	G	A	0.0389	0.0071	0.2088	0.0758	0.0059	0.2253
MPHOSPH6	16	82166375	rs7194734	T	C	0.0369	0.0060	0.1154	0.0655	0.0780	0.4775
ZNF208	19	22032639	rs8105767	A	G	0.0392	0.0054	0.0867	0.0586	0.1388	0.9426
RTEL1/STMN3	20	63638397	rs75691080	T	C	0.0671	0.0089	0.2854	0.0957	0.0029	0.3078
RTEL1	20	63660246	rs34978822	G	C	0.1397	0.0227	0.7018	0.2032	0.0006	0.0805
RTEL1/ZBTB46	20	63805045	rs73624724	T	C	0.0507	0.0074	0.1050	0.0837	0.2096	0.9926

[a]Summary statistics available from Li et al. (2020)

[b]β estimate for each variant. Multivariable models (two-sided logistic regression) included the variant of interest and controlled for significant principal components

[c]False discovery rate for each variant as calculated using GLIDE (Dai et al. (2018)). Five variants (rs4691895, rs7705526, rs34991172, rs228595, rs34978822) were detected to have evidence of pleiotropy (FDR <0.2) and removed from subsequent analyses

One variant was not imputed in our cohort: rs55749605 (Li et.al AAF = 0.58)

Supplemental Table 12. Distribution of measured telomere length by population characteristics

Characteristic	Mean (SD)	p-value[a]
Sex		0.2760
Male	0.34 (0.13)	
Female	0.35 (0.14)	
Age Quartile		0.0037
≤52	0.37 (0.16)	
53-58	0.33 (0.12)	
59-63	0.33 (0.12)	
≥64	0.33 (0.13)	
Genetic Ancestry Category		0.9020
EUR	0.34 (0.14)	
AFR	0.36 (0.14)	
ASN	0.33 (0.12)	
AFR and EUR	0.34 (0.11)	
ASN and EUR	0.37 (0.13)	
Other	0.32 (0.11)	
DNA Source		0.0031
Whole Blood	0.35 (0.13)	
PBMC	0.30 (0.13)	
Myelofibrosis Type		0.0016
Primary	0.35 (0.14)	
Secondary	0.32 (0.12)	
DIPSS at Transplant		0.7940
Low	0.38 (0.15)	
Intermediate 1	0.36 (0.15)	
Intermediate 2	0.32 (0.11)	
High	0.32 (0.14)	
Missing	0.35 (0.13)	
Time from diagnosis to transplant Quartile (months)		0.0171
≤9	0.38 (0.15)	
10-24	0.37 (0.14)	
25-88	0.34 (0.12)	
≥89	0.29 (0.10)	
Missing	0.28 (0.11)	

[a]One-way ANOVA

Source data are provided as a Source Data file

Supplemental Table 13. Population characteristics by autosomal mCA status

Characteristic	Autosomal Mosaicism Yes	Autosomal Mosaicism No	p-value[a]
N	671	241	-
DNA Source, N (%)			0.0183
Whole Blood	627 (93.44)	213 (88.38)	
PBMC	44 (6.56)	28 (11.62)	
Sex, N (%)			0.0804
Male	375 (55.89)	151 (62.66)	
Female	296 (44.11)	90 (37.34)	
Age, Mean (SD)	57.77 (9.48)	54.42 (12.53)	3.16×10^{-5}
Age Quartile, N (%)			0.0107
≤52	150 (22.35)	80 (33.20)	
52-58	171 (25.48)	54 (22.41)	
59-64	173 (25.78)	55 (22.82)	
>64	177 (26.38)	52 (21.58)	
Genetic Ancestry, Mean (SD)			0.4942
EUR	0.91 (0.23)	0.91 (0.23)	
AFR	0.05 (0.16)	0.06 (0.18)	
ASN	0.04 (0.16)	0.03 (0.14)	
Genetic Ancestry Category, N (%)			0.5402
EUR	600 (89.42)	212 (87.97)	
AFR	11 (1.64)	7 (2.90)	
ASN	18 (2.68)	4 (1.66)	
AFR and EUR	19 (2.83)	9 (3.73)	
ASN and EUR	13 (1.94)	7 (2.90)	
Other	10 (1.49)	2 (0.83)	
Myelofibrosis Type			0.0378
Primary	445 (66.32)	178 (73.86)	
Secondary	226 (33.68)	63 (26.14)	
DIPSS at Transplant, N (%)			0.0172
Low	46 (6.86)	27 (11.20)	
Intermediate 1	144 (21.46)	67 (27.80)	
Intermediate 2	188 (28.02)	50 (20.75)	
High	5 (0.75)	3 (1.24)	
Missing	288 (42.92)	94 (39.00)	
Time from diagnosis to transplant (months), Mean (SD)	67.02 (81.07)	56.40 (87.63)	0.0828
Measured Telomere Length, Mean (SD)			
Overall	0.33 (0.13)	0.37 (0.13)	7.93×10^{-5}
Whole Blood	0.34 (0.13)	0.38 (0.13)	5.73×10^{-4}
PBMC	0.26 (0.11)	0.36 (0.13)	8.97×10^{-4}

[a]Chi-square test for categorical variables, One-way ANOVA for continuous variables

mCA= mosaic chromosomal alteration

Source data are provided as a Source Data file

Supplemental Table 14. Distribution of measured telomere length by *JAK2*^{V617F} mutation status and autosomal mCA status

Subject Group	N	Mean (SD)	Median	IQR
No *JAK2*^{V617F} mutation or mCA	143	0.36 (0.13)	0.34	0.28-0.45
No mCA	241	0.37 (0.13)	0.35	0.28-0.45
No *JAK2*^{V617F} mutation	356	0.35 (0.15)	0.32	0.25-0.41
JAK2^{V617F} mutation	549	0.34 (0.13)	0.32	0.25-0.41
Any mCA	671	0.33 (0.13)	0.31	0.24-0.40
mCA over 9p24.1	368	0.32 (0.12)	0.30	0.24-0.39
Multiple mCAs	448	0.32 (0.13)	0.29	0.23-0.38
JAK2^{V617F} mutation and mCA over 9p24.1	358	0.32 (0.12)	0.30	0.24-0.39

mCA= mosaic chromosomal alteration

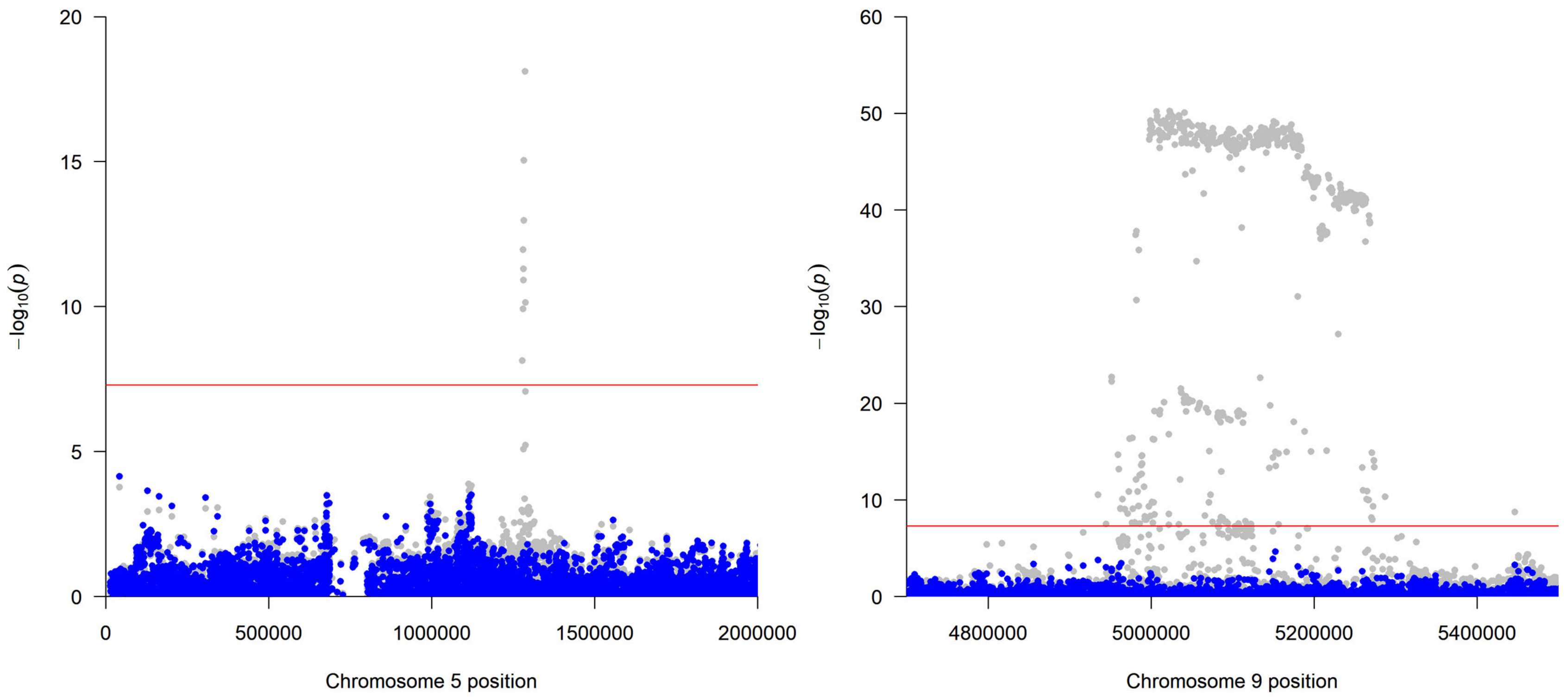

Supplemental Figure 1. Conditional genome-wide association analyses at the 5p15.33 and 9p24.1 regions. The overall $-\log_{10}$ P-values are plotted in gray (two-sided, logistic regression). The $-\log_{10}$ P-values after conditioning on the top tagging SNP in each region (rs7705526 at 5p15.33 and rs7851556 at 9p24.1) are plotted in blue (two-sided, logistic regression). The red line indicates the genome wide significance threshold (5×10^{-8}). No additional independent signals were identified in either region.

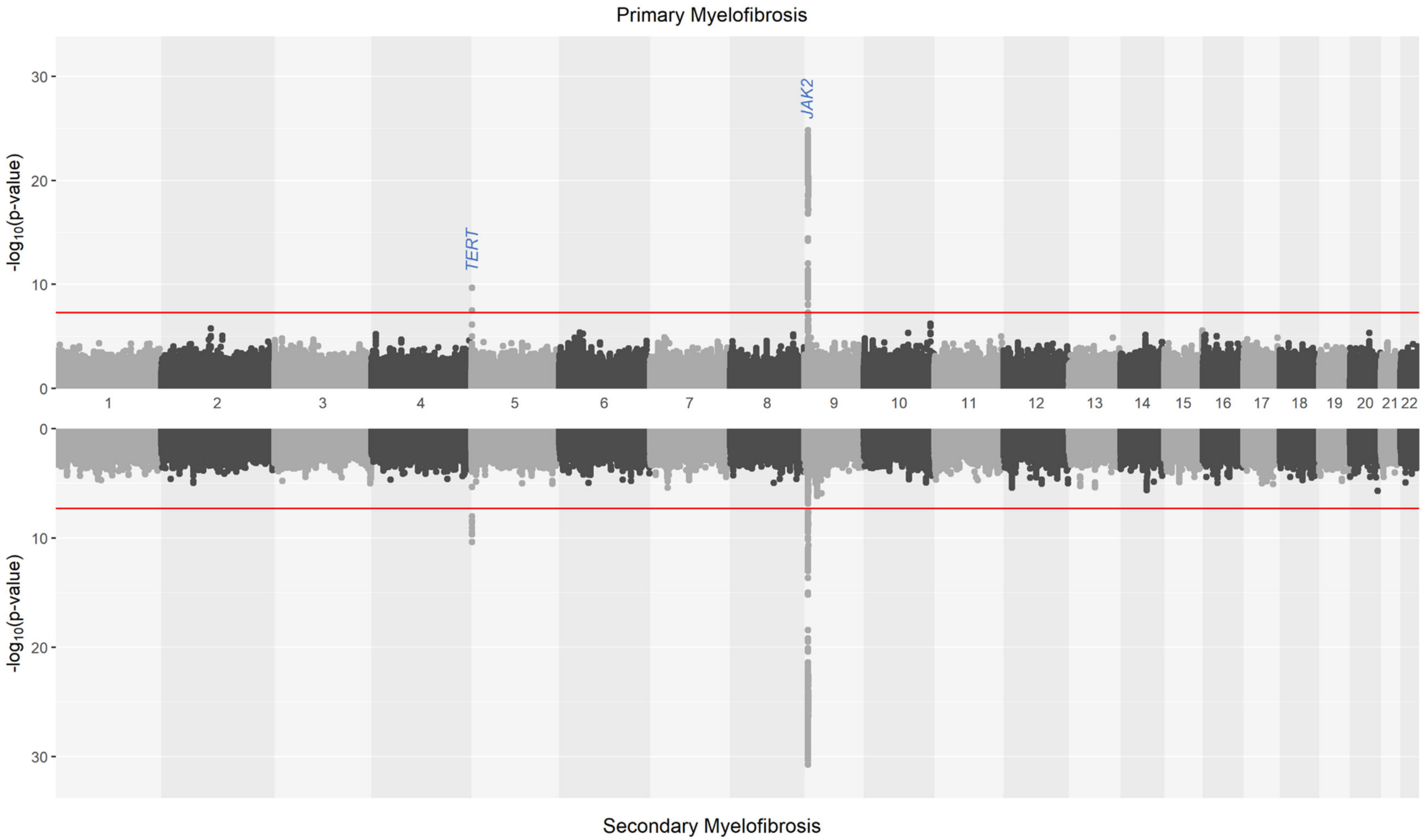

Supplemental Figure 2. Stacked Manhattan plots from the genome-wide association study stratified by primary (569 cases, 2,845 controls; Top plot) and secondary (258 cases, 1,290 controls; Bottom plot) myelofibrosis status. The association -$\log_{10}$ P-values are plotted for each tested genetic variant on the y-axis (two-sided, logistic regression) and chromosomal position on the x-axis. The nearest gene for each identified locus is labeled. The red line indicates the genome wide significance threshold (5×10^{-8}). Source data are provided as a Source Data file.

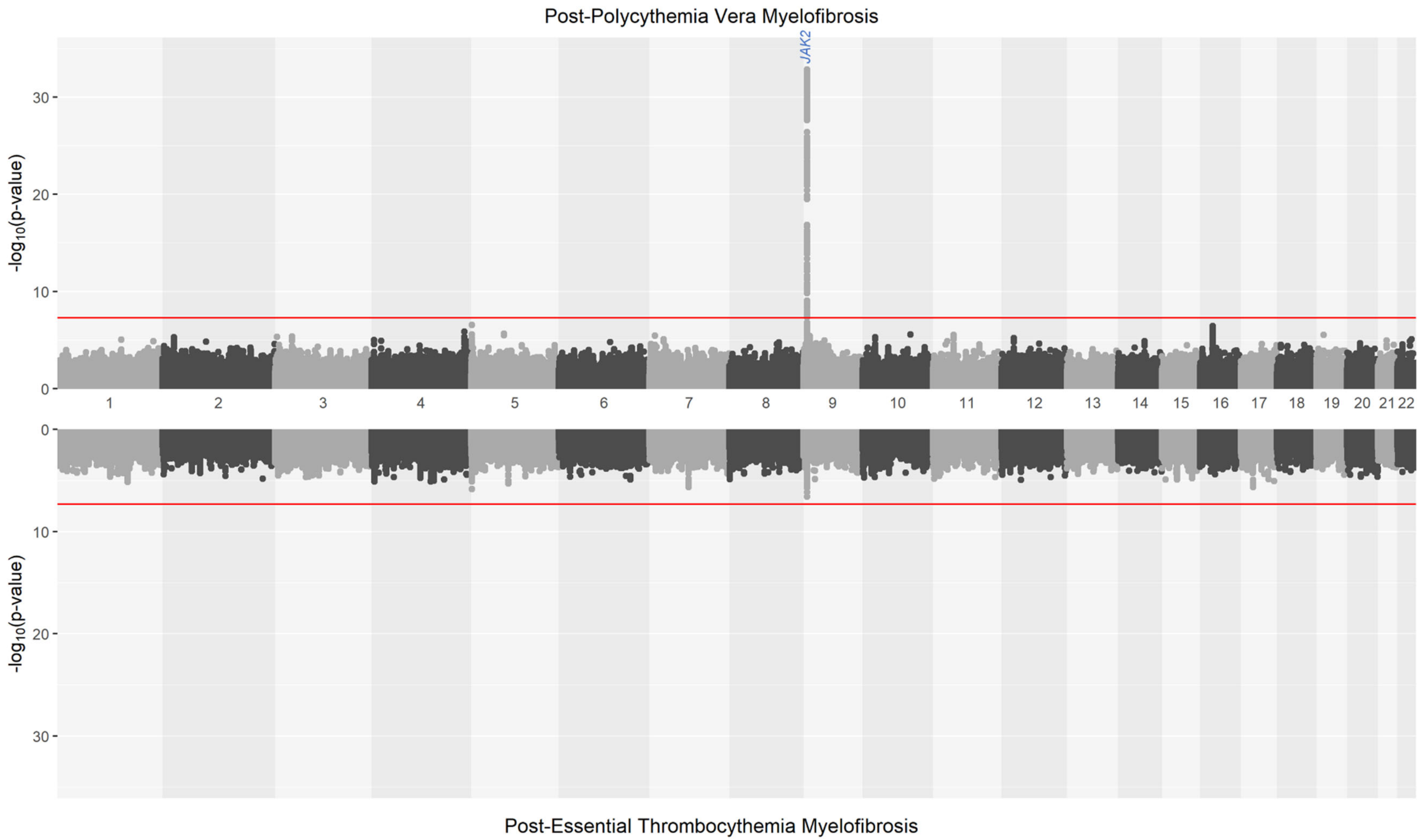

Supplemental Figure 3. Stacked Manhattan plots from the genome-wide association study stratified by post-polycythemia vera myelofibrosis (119 cases, 595 controls; Top plot) and post-essential thrombocythemia myelofibrosis (139 cases, 695 controls; Bottom plot). The association -$\log_{10}$ P-values are plotted for each tested genetic variant on the y-axis (two-sided, logistic regression) and chromosomal position on the x-axis. The nearest gene for each identified locus is labeled. The red line indicates the genome wide significance threshold (5×10^{-8}). Source data are provided as a Source Data file.

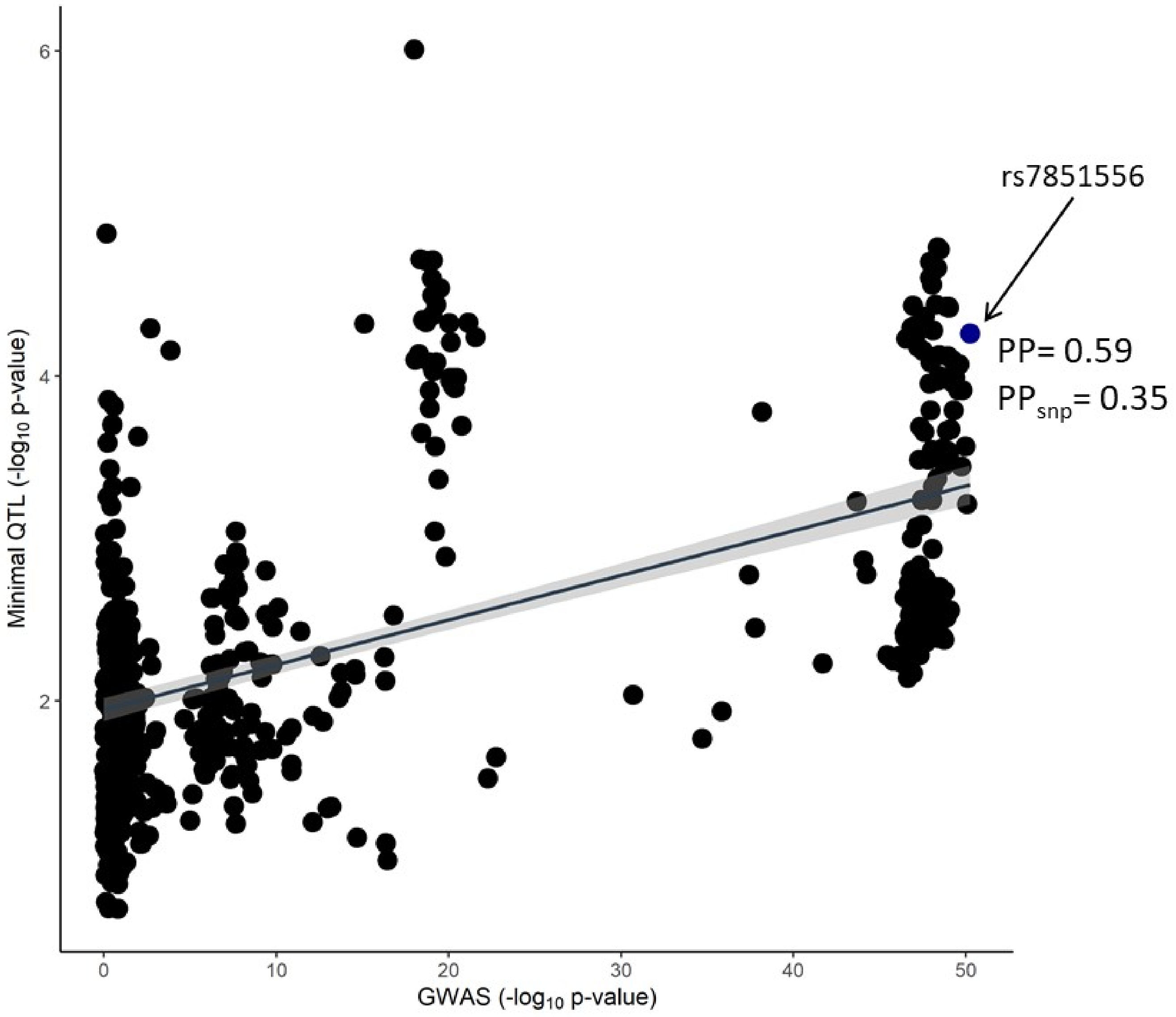

Supplemental Figure 4. Myelofibrosis GWAS colocalizing with whole blood eQTL at 9p24.1 (posterior probability (PP)= 0.59). The y-axis depicts minimal QTL -$\log_{10}$ P-values for each tested local SNP 100 kb upstream and downstream of the lead GWAS SNP (rs7851556). The x-axis depicts GWAS -$\log_{10}$ P-values for each SNP (two-sided, logistic regression). rs7851556 was associated with increased *JAK2* expression and explained 35% (PP_{SNP}= 0.35) of the shared colocalization signal. A linear model estimated trend line and calculated 95% confidence interval around the trend (shaded gray) are also plotted. Source data are provided as a Source Data file.

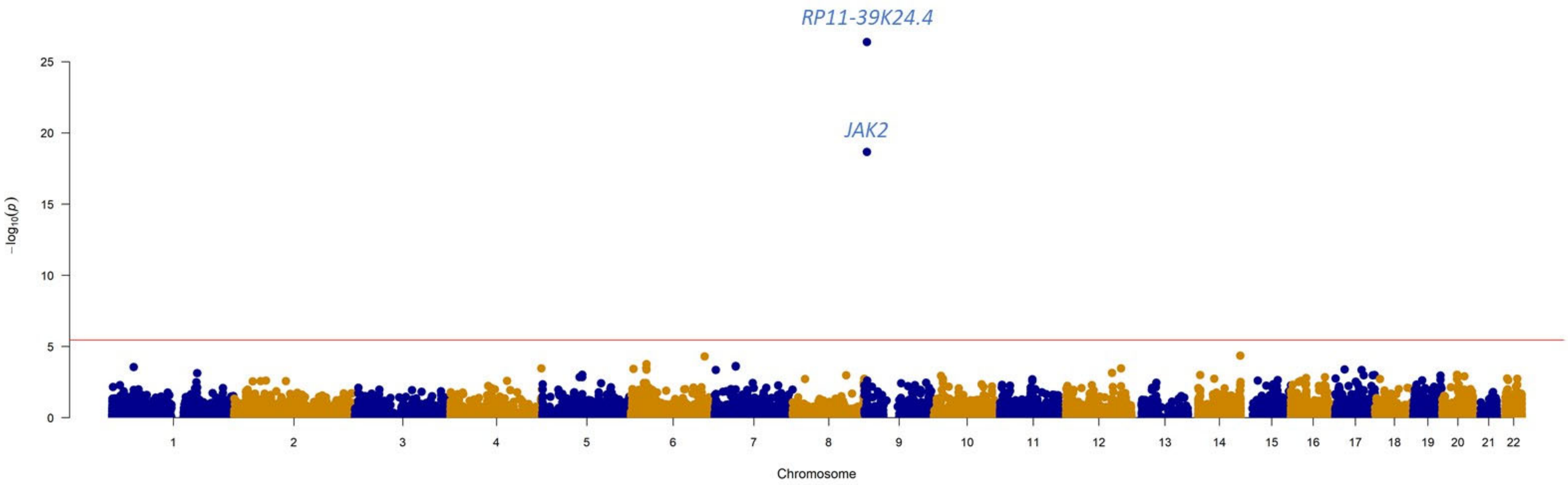

Supplemental Figure 5. Manhattan plot from the myelofibrosis transcriptome-wide association study using whole blood eQTL data. The association two-sided -$\log_{10}$ P-values are plotted for each imputed gene on the y-axis and chromosomal position on the x-axis. The red line indicates the Bonferroni-corrected level of significance (3.59×10^{-6}). Source data are provided as a Source Data file.

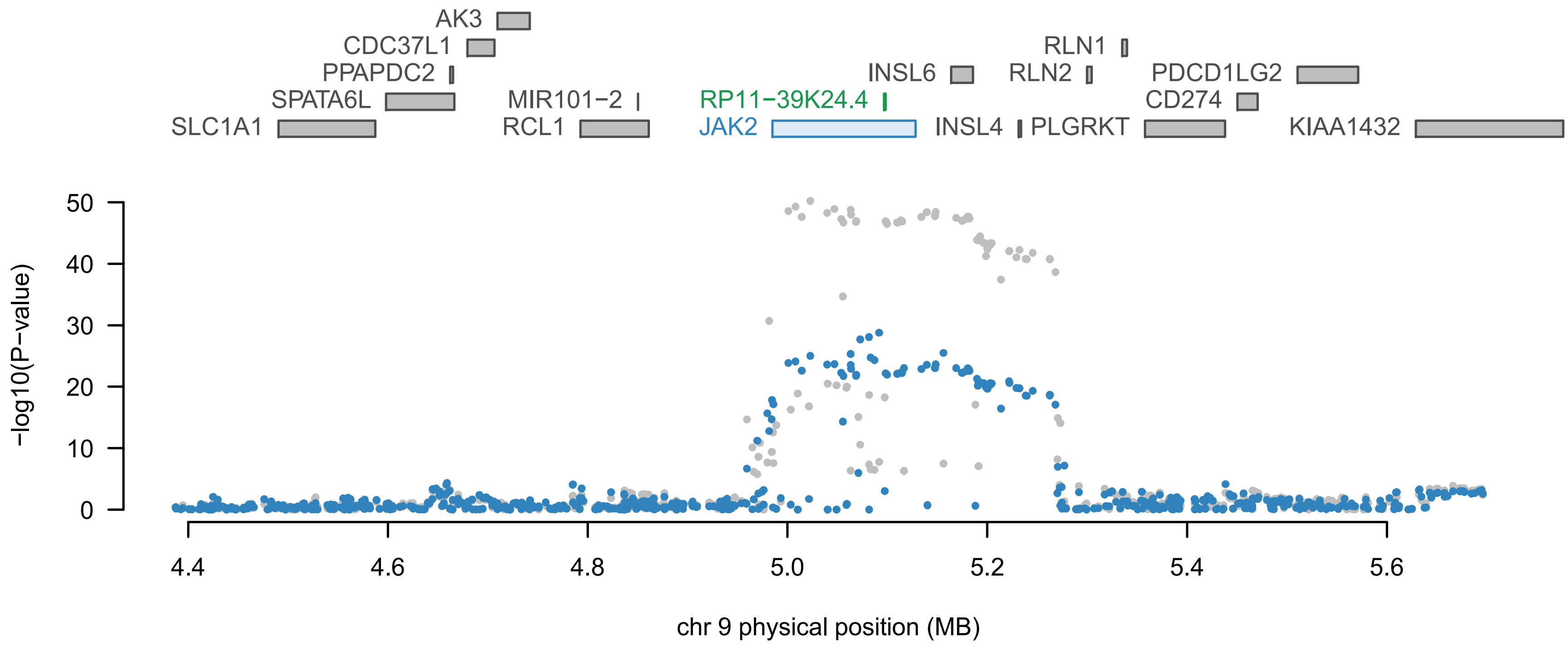

Supplemental Figure 6. TWAS using whole blood eQTL data identified two myelofibrosis-associated genes at the 9p24.1 region. The lead TWAS identified gene *RP11-39K24.4* is shown in green with the second significant gene *JAK2* in blue. All other annotated genes at this locus are given in gray. The Manhattan plot presents the myelofibrosis GWAS P-values before (gray) and after (blue) conditioning on *RP11-39K24.4* imputed gene expression (two-sided, logistic regression).

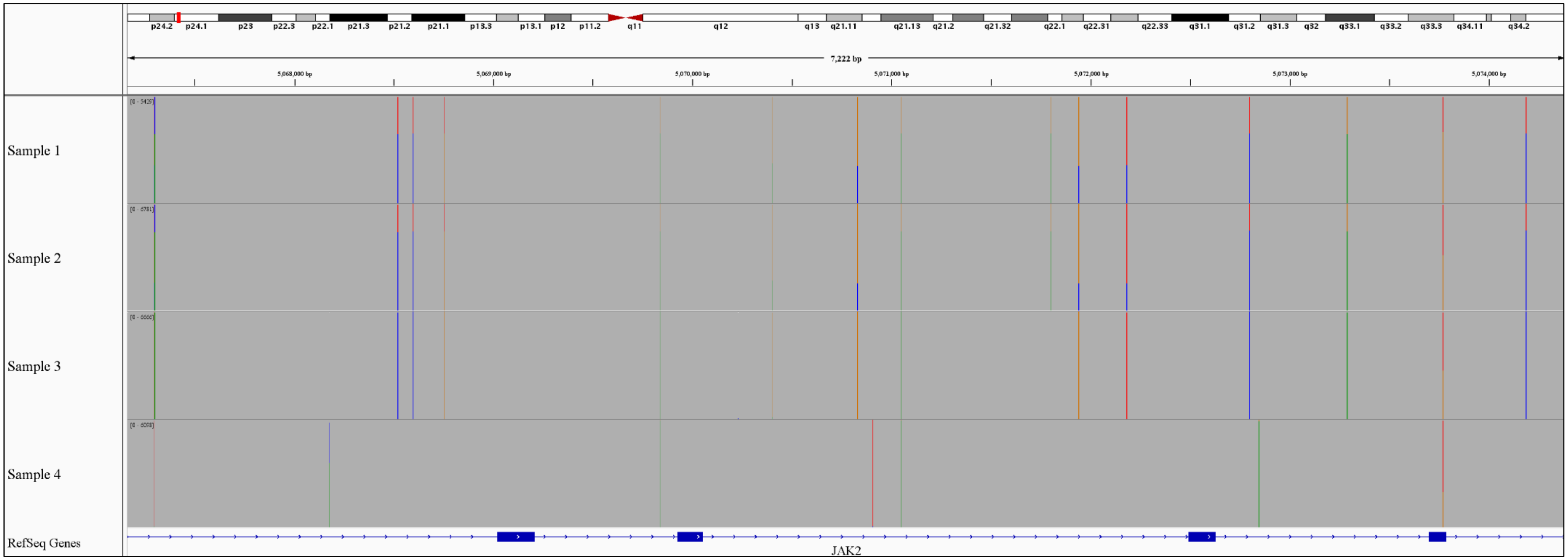

Supplemental Figure 7. IGV plot of four long-read PacBio sequencing samples demonstrating allelic imbalance of heterozygous variants. The colored bars represent the read count proportion of each base (A= green, C= blue, G= orange, T= red). Each sample has increasing cellular fraction (CF) of *JAK2* mosaic chromosomal alterations: Sample 1= 30% CF; Sample 4= 60% CF.

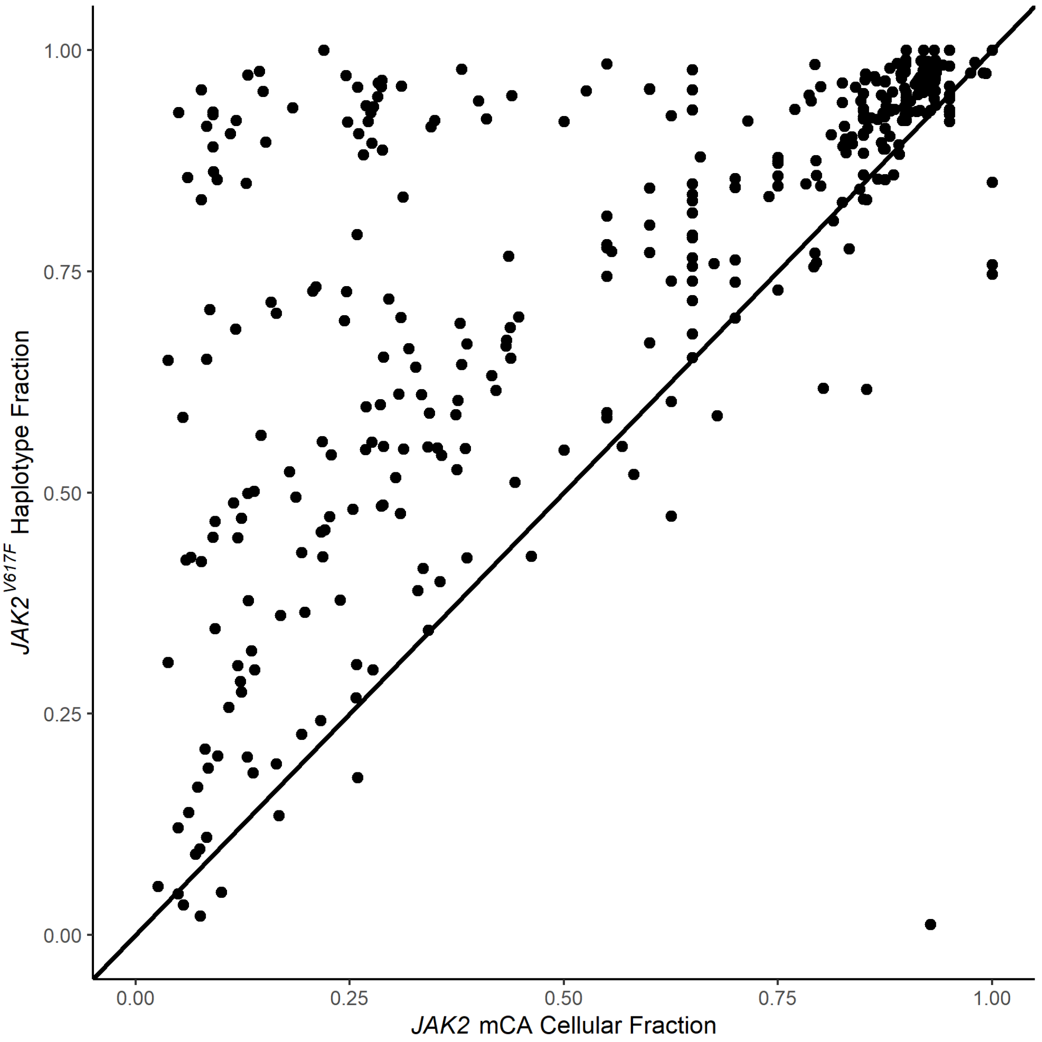

Supplemental Figure 8. Clonal evolution of *JAK2* mutations. The y-axis depicts *JAK2*^{V617F} haplotype fraction. The x-axis depicts *JAK2* mosaic chromosomal alteration (mCA) cellular fraction. *JAK2*^{V617F} haplotype fraction was larger than mCA cellular fraction in 323 (88.25%) individuals. Source data are provided as a Source Data file.

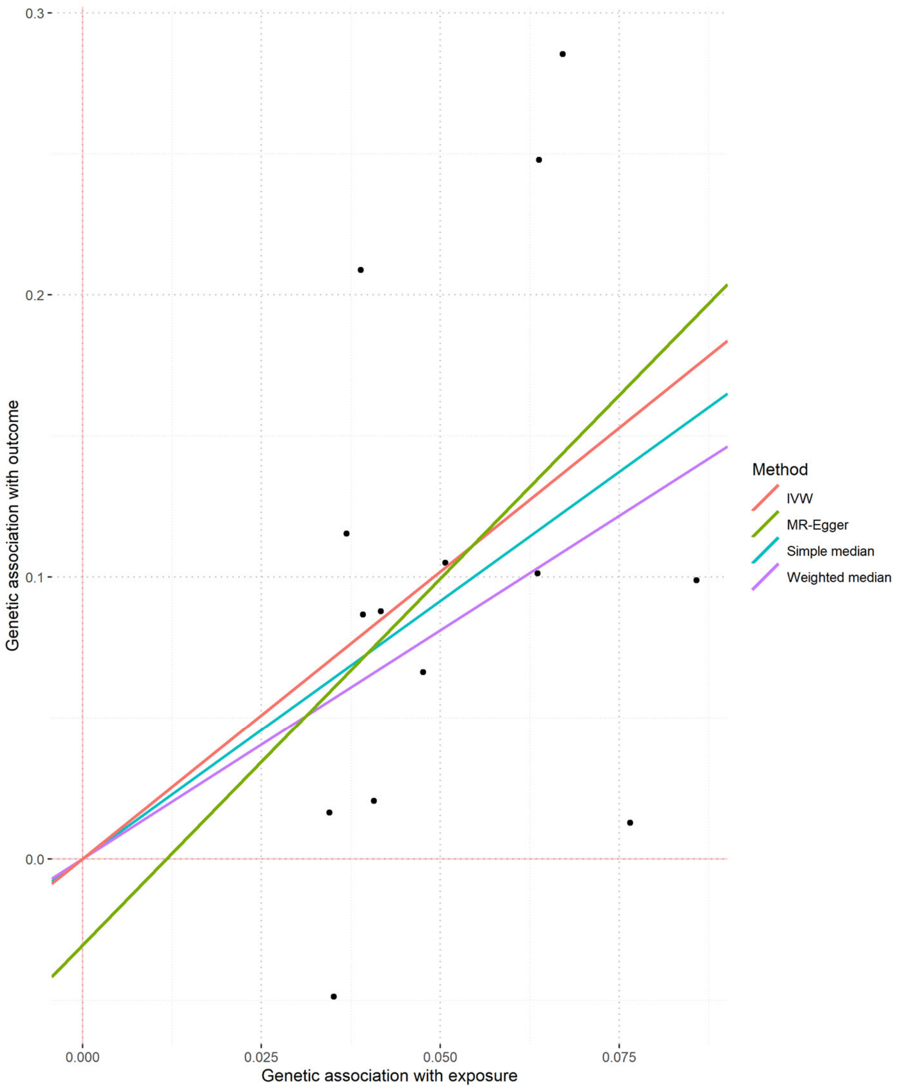

Supplemental Figure 9. Mendelian randomization (MR) estimates between telomere length-associated variants and myelofibrosis risk. The plot contains telomere length-associated variants from Li et al. (2020). Detailed MR results are presented in **Table 1**, all reported tests are two-sided. The MR-Egger intercept was non-significant (P= 0.65) after removing five variants detected to have evidence of pleiotropy (**Supplemental Table 11**).

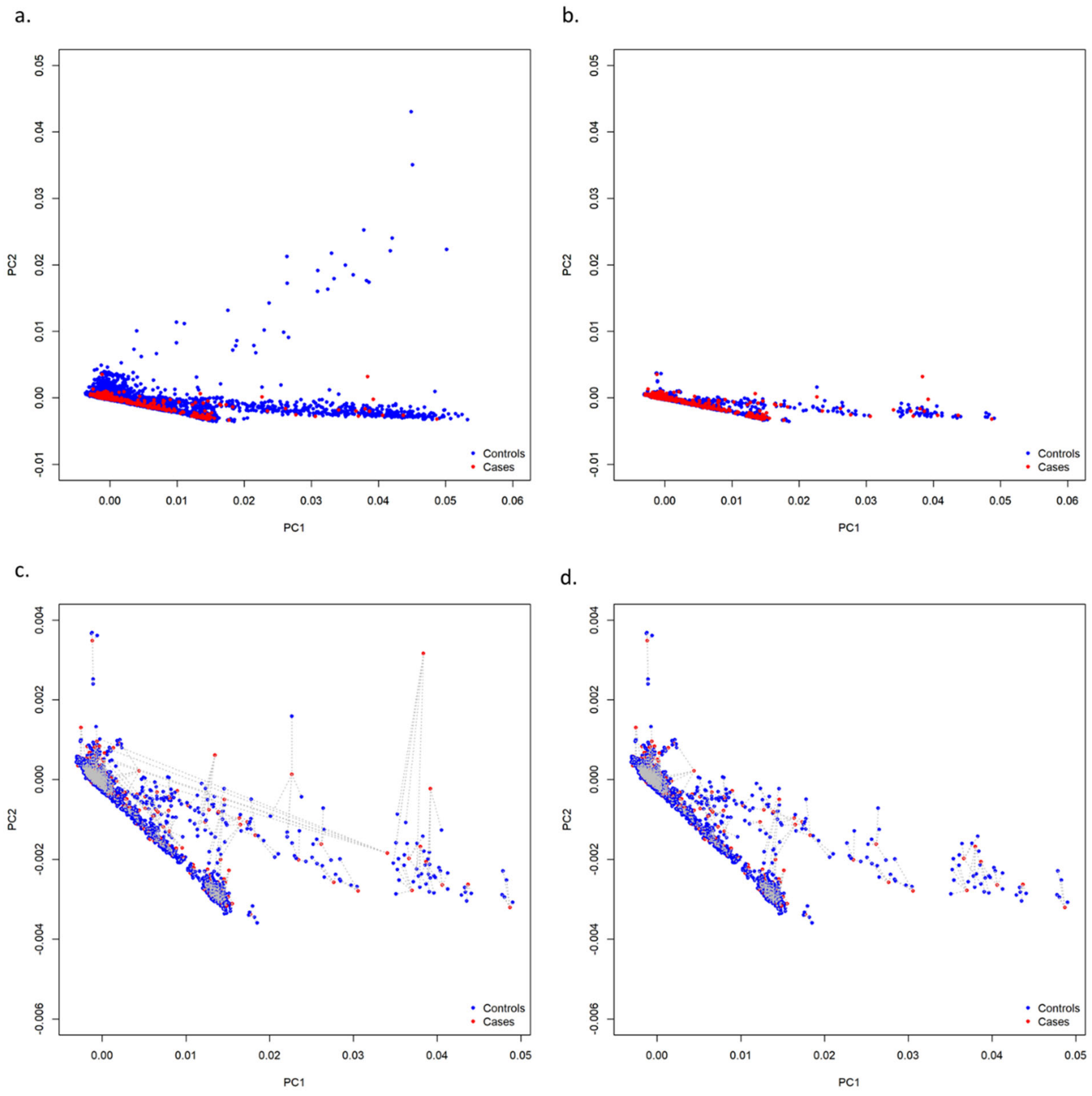

Supplemental Figure 10. Principal component (PC) matching of myelofibrosis cases (red) and PLCO controls (blue) for the genome wide-association study. In each plot, the x-axis is PC 1, and the y-axis is PC 2. Plot (**a**) depicts cases (N=833) and all potential controls (N= 56,929). (**b**) depicts cases (N=833) and 1:5 matched controls (N= 4,165). (**c**) depicts connected cases and matched controls. (**d**) depicts final matched sets after removing six cases found to have poorly matched controls. The final GWAS cohort contained 827 myelofibrosis cases and 4,135 genetically-matched controls. Source data are provided as a Source Data file.
