# Supplementary material for: Germline-somatic JAK2 interactions are associated with clonal expansion in myelofibrosis
Source: Nat Commun. 2022 Sep 8;13:5284. doi: 10.1038/s41467-022-32986-7 (PMC9458655; doi:10.1038/s41467-022-32986-7)
Supplement: Supplementary file 3 — Reporting Summary [file 41467_2022_32986_MOESM3_ESM.pdf]

nature portfolio

Corresponding author(s): Derek W. Brown, Mitchell J. Machiela

Last updated by author(s): 2022/08/17

Reporting Summary

Nature Portfolio wishes to improve the reproducibility of the work that we publish. This form provides structure for consistency and transparency in reporting. For further information on Nature Portfolio policies, see our Editorial Policies and the Editorial Policy Checklist.

Statistics

For all statistical analyses, confirm that the following items are present in the figure legend, table legend, main text, or Methods section.

n/a	Confirmed	
☐	☒	The exact sample size (*n*) for each experimental group/condition, given as a discrete number and unit of measurement
☒	☐	A statement on whether measurements were taken from distinct samples or whether the same sample was measured repeatedly
☐	☒	The statistical test(s) used AND whether they are one- or two-sided<br>*Only common tests should be described solely by name; describe more complex techniques in the Methods section.*
☐	☒	A description of all covariates tested
☐	☒	A description of any assumptions or corrections, such as tests of normality and adjustment for multiple comparisons
☐	☒	A full description of the statistical parameters including central tendency (e.g. means) or other basic estimates (e.g. regression coefficient) AND variation (e.g. standard deviation) or associated estimates of uncertainty (e.g. confidence intervals)
☐	☒	For null hypothesis testing, the test statistic (e.g. *F*, *t*, *r*) with confidence intervals, effect sizes, degrees of freedom and *P* value noted<br>*Give P values as exact values whenever suitable.*
☒	☐	For Bayesian analysis, information on the choice of priors and Markov chain Monte Carlo settings
☒	☐	For hierarchical and complex designs, identification of the appropriate level for tests and full reporting of outcomes
☒	☐	Estimates of effect sizes (e.g. Cohen's *d*, Pearson's *r*), indicating how they were calculated

Our web collection on statistics for biologists contains articles on many of the points above.

Software and code

Policy information about availability of computer code

Data collection	N/A- no software was used to collect data for this study.
Data analysis	LDSC(v1.0.1), SNPTEST(v2.5.6), SNPWEIGHTS(v2.1), PLINK(v1.9), R(3.6.3), Eagle(v2.4.1), MoChA(https://github.com/freeseek/mocha; version 2021-05-14), BAFSegmentation(v1.2.0), FUSION-TWAS (v1.0), eCAVIAR(v1.0), Hyprcoloc(v1.0), IGV(v2.11.9)

For manuscripts utilizing custom algorithms or software that are central to the research but not yet described in published literature, software must be made available to editors and reviewers. We strongly encourage code deposition in a community repository (e.g. GitHub). See the Nature Portfolio guidelines for submitting code & software for further information.

Data

Policy information about availability of data

All manuscripts must include a data availability statement. This statement should provide the following information, where applicable:

- Accession codes, unique identifiers, or web links for publicly available datasets
- A description of any restrictions on data availability
- For clinical datasets or third party data, please ensure that the statement adheres to our policy

Data from the genotyped and sequenced myelofibrosis individuals is available on dbGaP under accession number phs002635.v1.p1 [https://www.ncbi.nlm.nih.gov/projects/gap/cgi-bin/study.cgi?study_id=phs002635.v1.p1]. CIBMTR supports accessibility of research in accord with the National Institutes of Health (NIH) Data Sharing Policy and the National Cancer Institute (NCI) Cancer Moonshot Public Access and Data Sharing Policy. The CIBMTR only releases de-identified datasets that comply with all relevant global regulations regarding privacy and confidentiality. All relevant results and data used to produce the manuscript tables and figures are provided as a Source Data File. The mCA calls and phenotypic UK Biobank data used in this study, which were used under license, are available from: http://

www.ukbiobank.ac.uk/. Genotype data from the Prostate, Lung, Colorectal, and Ovarian (PLCO) Screening Trial is available on dbGaP under accession number phs001286.v2.p2 [https://www.ncbi.nlm.nih.gov/projects/gap/cgi-bin/study.cgi?study_id=phs001286.v2.p2].

Field-specific reporting

Please select the one below that is the best fit for your research. If you are not sure, read the appropriate sections before making your selection.

☒ Life sciences ☐ Behavioural & social sciences ☐ Ecological, evolutionary & environmental sciences

For a reference copy of the document with all sections, see nature.com/documents/nr-reporting-summary-flat.pdf

Life sciences study design

All studies must disclose on these points even when the disclosure is negative.

Sample size	A total of 937 individuals with myelofibrosis and available blood samples and clinical information from the Center for International Blood and Marrow Transplant Research (CIBMTR) database and repository.
Data exclusions	GWAS analyses were restricted to 827 individuals with European ancestry. Standard quality control checks were performed to ensure high completion rates (≥95%), no sample contamination, sex concordance, no unexpected duplicates or replicates, normal rates of heterozygosity, and no instances of high relatedness (IBD < 0.2).
Replication	Myelofibrosis is a rare disease and large well-characterized case series for replication are not available.
Randomization	This was a case-series and case-control study, so randomization is not part of the study design.
Blinding	All laboratory personnel were blinded to case control status for genotyping, targeted sequencing and telomere length assessment.

Reporting for specific materials, systems and methods

We require information from authors about some types of materials, experimental systems and methods used in many studies. Here, indicate whether each material, system or method listed is relevant to your study. If you are not sure if a list item applies to your research, read the appropriate section before selecting a response.

Materials & experimental systems

n/a	Involved in the study	
☒	☐	Antibodies
☒	☐	Eukaryotic cell lines
☒	☐	Palaeontology and archaeology
☒	☐	Animals and other organisms
☐	☒	Human research participants
☒	☐	Clinical data
☒	☐	Dual use research of concern

Methods

n/a	Involved in the study	
☒	☐	ChIP-seq
☒	☐	Flow cytometry
☒	☐	MRI-based neuroimaging

Human research participants

Policy information about studies involving human research participants

Population characteristics	This study utilized blood samples and clinical information from the Center for International Blood and Marrow Transplant Research (CIBMTR) database and repository. The majority of myelofibrosis patients in our study were male (58.06%) and had DNA collected at an average age of 56.9 years (median=58.4, IQR=52.3-63.9). Most patients were primary (68.84%) myelofibrosis, with intermediate 1 or 2 disease (49.30%) based on the Dynamic International Prognostic Scoring System score. The average time from diagnosis to transplant in the full cohort was 63.5 months (median= 25.1, IQR=9.0-88. 3).
Recruitment	Patients eligible for inclusion were those who underwent hematopoietic cell transplantation (HCT) for primary idiopathic or secondary MF between 2000 and 2016 and have a pre-HCT blood sample available for genomic analysis. Blood samples, from either whole blood or peripheral blood mononuclear cells (PBMCs), were collected within 30 days before administering HCT conditioning regimen. In total, 937 MF patients met the criteria for inclusion in our study, of which 863 (92.1%) contributed whole blood and 74 (7.9%) PBMCs.
Ethics oversight	The study was approved by the National Marrow Donor Program institutional review board. All patients provided written informed consent for the research use of their samples and clinical data.

Note that full information on the approval of the study protocol must also be provided in the manuscript.
